# Microwave-Assisted Solvent-Free Acetylation of Cellulose with Acetic Anhydride in the Presence of Iodine as a Catalyst

**DOI:** 10.3390/molecules14093551

**Published:** 2009-09-11

**Authors:** Jing Li, Li-Ping Zhang, Feng Peng, Jing Bian, Tong-Qi Yuan, Feng Xu, Run-Cang Sun

**Affiliations:** 1 College of Material Science and Technology, Beijing Forestry University, Beijing 100083, China; 2 State Key Laboratory of Pulp and Paper Engineering, South China University of Technology, Guangzhou 510640, China

**Keywords:** cellulose, acetylation, microwave irradiation, iodine, solvent-free

## Abstract

In this work an optimization of the solvent-free acetylation of cellulose with acetic anhydride under microwave heating with iodine as a catalyst was performed. The optimized parameters included the microwave irradiation power from 300 W to 800 W, the reaction time between 5 to 40 min, the reaction temperature from 80 to 130 °C, and the amount of iodine from 1 to 15 mol%. The extent of the acetylation was measured by yield and the degree of substitution (DS), which was determined by a back-titration method. Acetylated cellulose was characterized by FT-IR, CP/MAS ^13^C-NMR, WRXD, and thermogravimetric analysis. The results showed that within the range of catalyst amounts studied, the DS increased as the amount of iodine used increased, however, it was barely affected by microwave output. It was also found that the reaction time and temperature had an active influence on the extent of acetylation, however, this did not mean that at the higher temperature a better acetylation of cellulose would be obtained. The optimal reaction time and temperature found in this work were 30 min and 130 °C.

## 1. Introduction

Environmental concerns such as pollution and decreases in natural resources, have led in recent years to an increased demand for renewable materials. Due to its non-toxic, renewable, biodegradable and readily modifiable properties, cellulose, the major constituent of all plant materials, which is a homopolymer composed D-glucopyranose units linked by β (1→4) glycosidic bonds [1], is attractive as a sustainable source of materials for industrial processes. Cellulose acetate (CA), one of the most important cellulose derivatives, has been utilized in a vast array of applications such as coatings, textile fibers, consumer products, filtration membranes, composites, laminates, and medical and pharmaceutical products [2,3,4,5]. Commercially, the largest amount of cellulose acetate is produced by the so-called “acetic acid process”. In this heterogeneous process, the cellulose is first swelled in acetic acid and then acetylated with acetic anhydride in the presence of sulfuric acid or perchloric acid as catalysts. Despite the advantages of low cost and high productivity, serious degradation of the cellulose and hydrolysis of cellulose acetate inevitably occur because of the water and remaining acid catalysts in the quenching step. In a word, even a wide availability of CA and a relatively mature industrial manufacture, its synthesis methods still need further investigation.

As is known, for esterification of cellulose, the common process is the reaction without solvent, because the solvent will reduce the reaction rate by dilution of modifiers, and use of a solvent would require complicated separation procedures to recover the chemicals after the reaction, which makes the process undesirable by increasing the production costs. In addition, organic solvents are often harmful to humans and environment. Therefore, a solvent-free version is preferable to eliminate the use of organic solvents [6]. In addition, microwave irradiation has been proven to be rapid, uniform and efficient, and to easily reach particles inside [7,8,9]. It offers an advantage over conventional techniques by reducing the required amount of solvent, the amount of waste produced, and the reaction time. In previous reports, a number of different polysaccharide derivatives have been synthesized with the aid of microwave irradiation in solvent-free or aqueous-based reaction systems [10,11,12,13]. Especially, as one of the power techniques of non-contact heating, microwave irradiation heating has been used for reacting, heating and drying cellulose materials and in the dyeing process of cellulose fabrics [7]. Generally speaking, the reaction is accelerated under microwave conditions mainly due to the speed with which a mixture can be heated and the high temperatures easily obtainable in vessels. In this study, one of the goals was to explore the effects of microwave heating on acetylation of cellulose with acetic anhydride in a solvent-free system, and to optimize the parameters of the microwave irradiation for obtaining relatively high DS values and yields and greatly reduced reaction times. Moreover, acetic anhydride was chosen due to the fact that acetic anhydride, unlike higher anhydrides, is a liquid thus making a solvent-free system possible. Energy is readily transferred by the microwave irradiation to the highly polar molecules of acetic anhydride through the characteristic dipolar activation of microwave heating [14]. In addition, among a variety of catalysts which have been chosen to catalyze the acetylation of cellulose, for instance sulfuric acid, perchloric acid, pyridine, triethylamine (TEA) and so on, iodine has been taken into account as a strong catalyst, and it has been used in various organic synthesis [17,18,19], as a novel, convenient, efficient, and cost-effective catalyst. During recent years, there have been many reports about the applications of iodine as a Lewis acid catalyst for esterification of polysaccharides like cellulose, starch, chitosan and so on [16,20,21,22,23,24].

**Figure 1:**
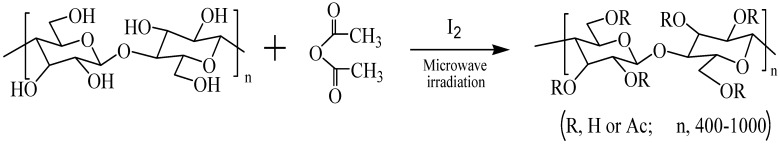
Modification of cellulose to cellulose acetate, R represents for H or Ac, n ranges from 400 to 1,000.

In the present work, all the merits of the acetylation of cellulose were combined, and a perfect reaction was anticipated, which is the microwave-assisted solvent-free acetylation of cellulose with acetic anhydride in the presence of iodine as a catalyst (Figure 1). In addition, our objective in this work was to explore the effects of reaction temperature, reaction duration, microwave irradiation power and iodine concentration on the extent of acetylation, in order to find the optimal reaction conditions to produce cellulose acetates in a more economical and environmentally friendly way by minimizing the energy consumption, the amount of solvent and catalyst, and especially the toxic by-products produced. All the samples were characterized by Fourier Transform infrared (FT-IR) and solid-state cross-polarization magic angle spinning carbon-13 nuclear magnetic resonance (CP/MAS ^13^C-NMR) spectroscopy, wide-angle X-ray diffraction (WXRD), and thermogravimetric analysis.

## 2. Results and Discussion

### 2.1. Optimization of the microwave irradiation

The microwave irradiation was optimized using the following process: 0.4075 g cellulose, 10 mL acetic anhydride and 5 mol% iodine (the molar ratio of I_2_/AGU) were mixed in a 50 mL three-necked flask fitted with a mechanical stirrer. Different reaction times (t), temperatures (T) and power outputs (P) were used to irradiate the mixture. The DS and yield were determined for each product (Table 1). The mixtures were heated at 120 °C for 15 min, the samples (sample 1-sample 6) were acetylated by increasing the power outputs from 300 W to 800 W, respectively. It was found that the DS and yield of sample 2 were higher than for other samples. Therefore, the irradiation power at 400 W was selected. Then, under the fixed conditions of output power at 400 W and reaction time for 15 min, when the reaction temperature was changed from 80 °C to 130 °C, the DS and yield of the corresponding samples increased. Evidently, when the reaction temperature was 130 °C, the highest DS and yield of 1.6 and 25% were obtained. Fixing the temperature (130 °C) and output of microwave irradiation (400 W), changing the reaction time from 5 to 40 min was considered. Upon increasing the reaction time from 5 to 40 min, the DS and yield of cellulose acetate increased correspondingly. However, considering the degradation of cellulose and hydrolysis of cellulose acetate at long reaction times [15], the condition of sample 17 was considered optimal. Therefore, the optimum condition was that the cellulose was acetylated at 130 °C for 30 min with irradiation power 400 W.

Meanwhile, the results clearly indicated that irradiation power had a little effect on the reaction, while reaction time and temperature played the important roles. In general, as weight increase is due to a balance of water lost and addition of acetyl groups, the DS and yield increased when the reaction time was extended and the temperature increased. It seems that the diffusion mechanisms play an important part in making inner OH groups available to the reaction with acetic anhydride. It was proposed that at the beginning of the reaction, the reaction was heterogeneous, and OH groups on the surface of solid cellulose will be preferentially acetylated and then, as the acetylation progressed, the acetylated cellulose gradually dissolved in the reaction medium and the remaining unreacted hydroxyls will be acetylated subsequently [16]. In addition, the high temperature made the swelling ability of cellulose and the diffusion rate of acetic anhydride and iodine increase significantly. However, the higher temperature and longer time will result in the hydrolysis of the ester groups and/or the decomposition of cellulose backbones.

### 2.2. Effects of iodine quantities

**Figure 2:**
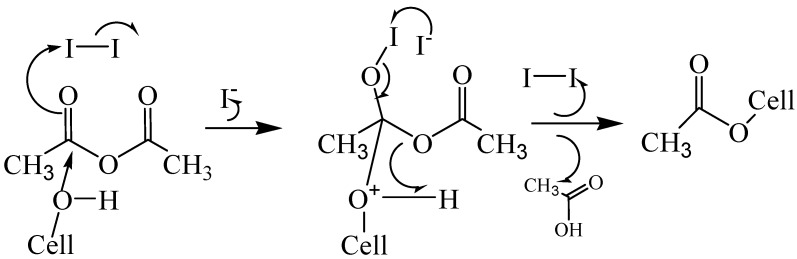
Mechanism of cellulose (Cell-OH) acetylation using iodine as a catalyst (based on Biswas *et al.* [23]).

A series of different concentrations of iodine (1, 3, 5, 8, 10, and 15 mol%) was added to a mixture which contained 0.4075 g cellulose and 10 mL acetic anhydride in a 50 mL three-necked flask fitted with a mechanical stirrer from sample 19 to sample 24. The mixtures were treated under the optimum conditions in which the cellulose samples were acetylated at 130 °C for 30 min under 400 W microwave power. The DS and yield are shown in Table 2. It has been previously reported that the initial step in the mechanism for the reaction of acetic anhydride with hydroxyl groups involves a nucleophilic attack on the acyl carbon center of the acetic anhydride molecule by a lone pair of the alcoholic (or phenolic) hydroxyl group followed by subsequent loss of acetic acid to generate the ester [25,26]. Cellulose can be regarded as a polyalcohol with the typical reactions of an alcohol, and iodine as a Lewis acid catalyst, activates the carbonyl carbon of acetic anhydride making the latter more reactive (Figure 2); this acetic anhydride-iodine combination works only in the absence of a solvent [23].

When cellulose was heated with a mixture of acetic anhydride and iodine under microwave irradiation, the acetylation occurred easily. As shown in Table 2, the DS and yield of the products increased with the increasing of the quantities of iodine, which is resulted by the fact that in the condition of a great excess of acetic anhydride, more acetic anhydride-iodine intermediates have been produced while increasing the amounts of iodine, and thus cellulose was more easily acetylated. With an increment in the amount of iodine from 1 to 15 mol%, the DS and yield of products increased up to 2.8 and 78%. For sample 19, the iodine catalysis still worked, but the degradation of cellulose dominated in the acidic solution under the microwave irradiation, which led to the observed weight loss of the products.

### 2.3. FT-IR spectra

**Figure 3:**
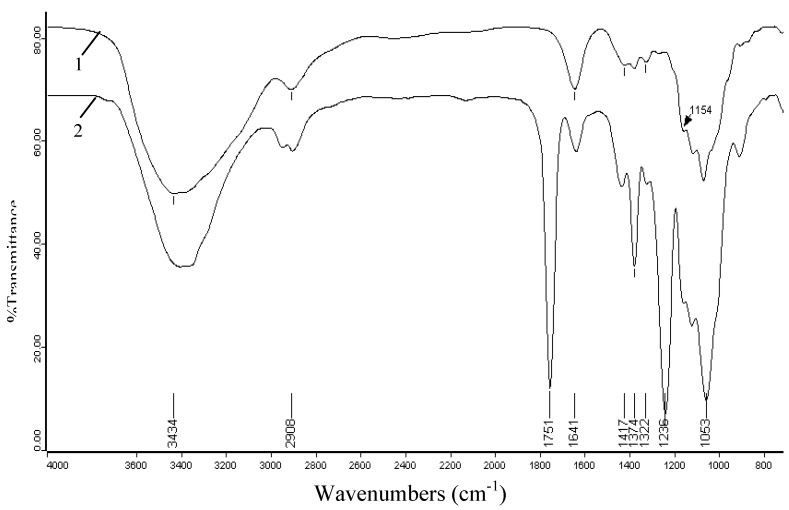
FT-IR spectra of unmodified cellulose (spectrum 1) and acetylated cellulose sample 17 (spectrum 2) prepared at 130 °C for 30 min with output 400 W using 5 mol% iodine as a catalyst.

The most convenient method for the elucidation of structural features of cellulosic production is FT-IR spectroscopy. Figure 3 illustrates the FT-IR spectra of unmodified cellulose (spectrum 1), and acetylated cellulose (spectrum 2, sample 17). The absorbances at 3,434, 2,908, 1,641, 1,374, 1,154, and 1,053 cm^-1^ in spectrum 1 are associated with native cellulose. A strong band at 3,434 cm^-1^ is attributed to the stretching of hydroxyl groups. The absorption at 2,908 cm^-1^ originates from the C-H stretching. The band at 1,641 cm^-1^ arises from the H-O-H bending of the absorbed water. The peak at 1,374 cm^-1^ corresponds to the O-H bending and that at 1,154 cm^-1^ is attributed to the C-O antisymmetric bridge stretching. A strong band at 1,053 cm^-1^ is due to the C-O-C pyranose ring skeletal vibration. Obviously, there was little lignin in the raw sample, which could be confirmed from the small absorbances at 1,322 and 1,417 cm^-1^ corresponding to the C-O stretching of the syringyl ring and aromatic skeletal vibrations [27]. In addition, the noncellulosic polysaccharides were almost completely eliminated, as indicated by the absence of a peak at 1,210 cm^-1^ [28].

**Figure 4:**
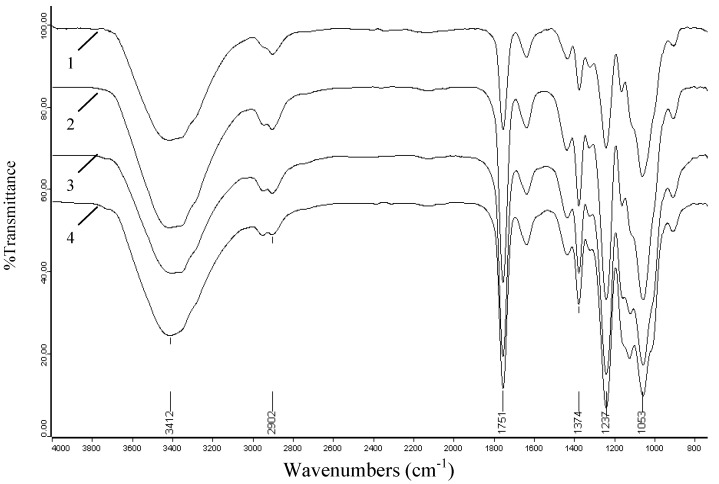
FT-IR spectra of acetylated cellulose samples prepared at different reaction times: 5 min (spectrum 1, sample 13), 20 min (spectrum 2, sample 15), 30 min (spectrum 4, sample 17), 40 min (spectrum 3, sample 18).

**Figure 5:**
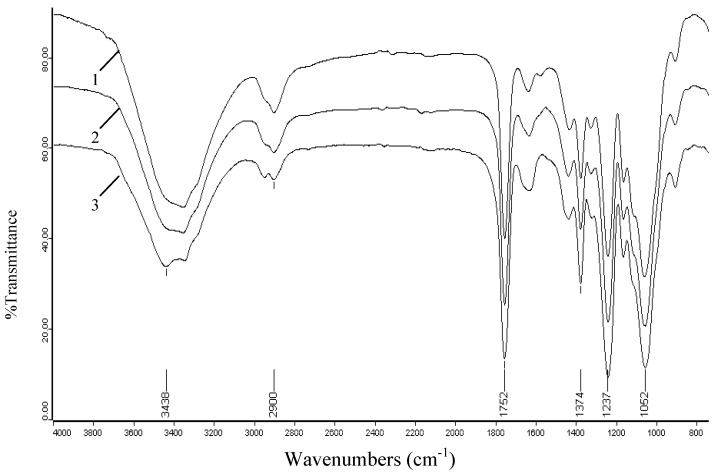
FT-IR spectra of acetylated cellulose samples prepared at different reaction temperatures: 90 °C (spectrum 1, sample 8), 110 °C (spectrum 2, sample 10), 130 °C (spectrum 3, sample 12).

**Figure 6:**
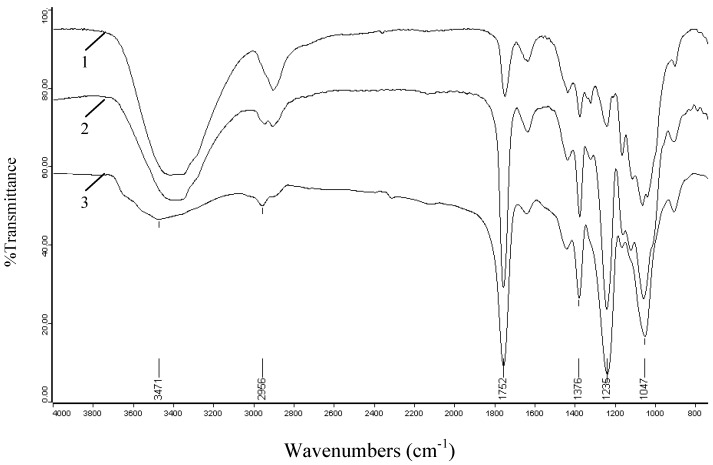
FT-IR spectra of acetylated cellulose samples prepared at different iodine concentrations: 1 mol% (spectrum 1, sample 19), 5 mol% (spectrum 2, sample 21), 15 mol% (spectrum 3, sample 24).

In comparison, the spectrum of acetylated cellulose shows obvious evidence of acetylation with three important ester bonds appearing at 1,751, 1,374, 1,236 cm^-1^ which belong to carbonyl C=O stretching of ester, C-H stretching in –O(C=O)-CH_3_, and C-O stretching of acetyl group, respectively. Another important aspect observed in the acetylated cellulose spectrum is the decreasing absorption intensity of the peak at 3,434 cm^-1^ assigned to the stretching vibrations of the O-H when compared to native cellulose. This decreasing trend occurs because the O-H is substituted by acetyl groups in the reaction and this decrease also further proves the happening of acetylation. In addition, the lack of peak in the region 1,840-1,760 cm^-1^ in spectrum 2 implied that the product is free of unreacted acetic anhydride. The absence of absorption at 1,700 cm^-1^ for a carboxylic group indicated that the product is also free of any acetic acid byproduct. The effects of reaction time, reaction temperature, and iodine concentrations on the extent of acetylation were also investigated by the peaks intensity of acetylated cellulose samples, and their spectra are given in Figure 4, Figure 5 and Figure 6.

### 2.4. Thermal analysis

**Figure 7:**
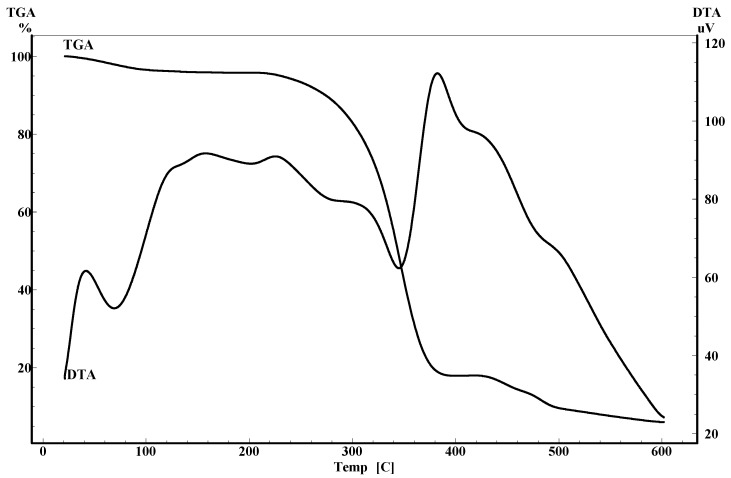
The differential thermal analysis and thermogravimetry analysis of cellulose.

**Figure 8:**
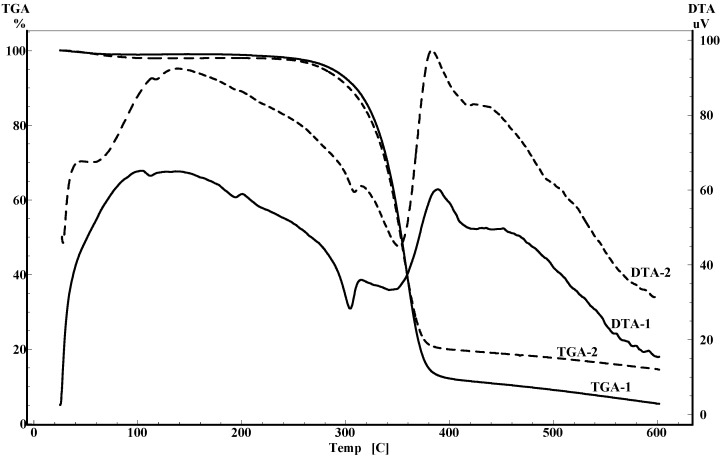
The differential thermal analysis and thermogravimetry analysis of acetylated cellulose sample 22 (TGA-1, DTA-1) and 7 (TGA-2, DTA-2).

The thermal properties of unacetylated cellulose and acetylated cellulose samples were examined in DTA and TGA studies in the temperature ranging from room temperature to 600 °C at a rate of 10 °C/min under nitrogen flow. The unacetylated cellulose is shown in Figure 7 and the acetylated samples 22 (thermogram 1) and 7 (thermogram 2) are illustrated in Figure 8. In Figure 7, from room temperature to 120 °C the TGA thermogram of unacetylated cellulose displayed a minor weight loss which is attributed to water desorption, which can also be explained by the endothermic event observed between 40 °C to 120 °C in DTA curve, however, this minor loss of weight is not obvious for the two acetylated cellulose samples 22 (thermogram 1) and 7 (thermogram 2). That is to say, the acetylated samples are more hydrophobic than the native cellulose. This result also can be proved by the fact that the endothermic peaks of DTA thermograms for samples 22 and 7 were much smaller than the endothermic peak of unacetylated cellulose. The onset thermal degradation temperatures were considered to start at 296 °C for unacetylated cellulose (Figure 7), 329 °C for sample 604 (Figure 8, thermogram 1), and 322 °C for sample 7 (Figure 8, thermogram 2), and 50% weight loss of native cellulose occured at 343 °C, while another two acetylated samples both happened at 354 °C. This increasing trend of decomposition temperature indicated that the thermal stability of the acetylated cellulose is higher than that of untreated cellulose. In addition, the endothermic peaks between 300 °C to 400 °C for three samples could be caused by decomposition, which was confirmed in TGA studies by the fast weight loss of the samples (Figure 7 and Figure 8). Near 400 °C, there are large exothermic peaks for the three samples in DTA curves, which related to the crystal structure of the native cellulose, and the peak of the cellulose is largest while the peak of the sample 22 is the smallest, this fact indicated that the degree of crystallinity for acetylated cellulose is lower than the native cellulose, and the degree of crystallinity decreased with the increase of the extent of acetylation.

### 2.5. WAXD spectra 

**Figure 9:**
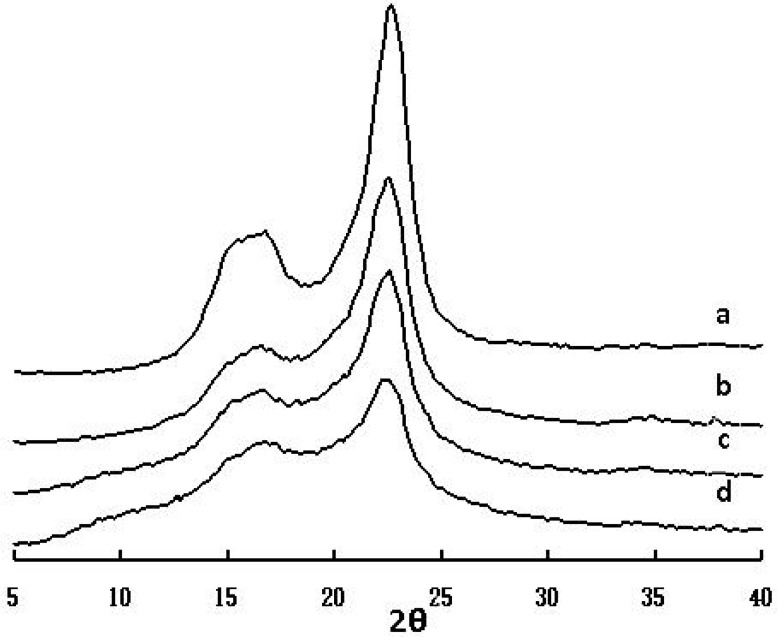
Wide-angle X-ray diffraction curves of native cellulose (spectrum a) and acetylated cellulose (sample 7, spectrum b; sample 11, spectrum c; sample 22, spectrum d).

**Figure 10:**
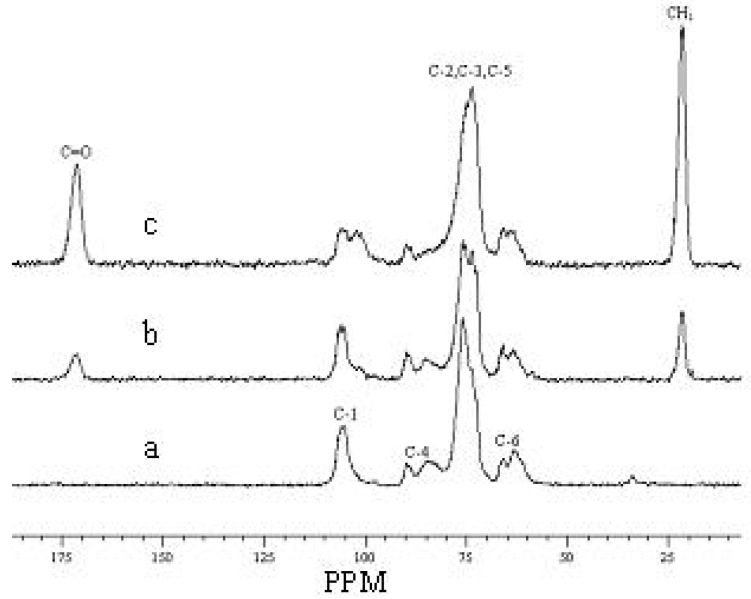
The CP/MAS ^13^C-NMR spectra of native cellulose (spectrum a) and acetylated cellulose sample 7 (spectrum b) and 22 (spectrum c).

The XRD results are presented in Figure 9 for the native sample and acetylated cellulose products. XRD spectra can display the changes in the crystallinity and structure of the samples after the acetylation. The native sample peaks localize at around 16.5 and 22.5 are assigned to the cellulose I in Figure 10, because the 22.5 is contributed to the 002 plane and 16.5 is due to the 101 and 10-1 plane. It is easily observed that the strength of signal for 002 plane is larger, while the peaks from 101 and 10-1 planes had diffusion phenomenon which resulted in overlapping. This may be due to the fact that the microcrystal of *Caragana korshinskii* cellulose is microscopic and during the preparation process the cellulose was partly swelled in acetic anhydride. All of the diffractograms presented a maximum located at 22.5; this maximum is found in all polymers which correspond to the amorphous region of the material, were known as van der Waals halo or amorphous halo, and another maximum around 17.0 was considered as the crystalline form [29].

Figure 9 displays that acetylated cellulose have a low degree of crystallinity, the reduction of crystallinity compared with the original cellulose was due to the substitution of the hydroxyl groups by acetyl groups that are the larger size groups, which broke the inter-and intra-molecular hydrogen bonds of cellulose, with the increasing number of hydroxyl groups being substituted, the crystallinity decrease. In addition, the X-ray diffraction grams of acetylated cellulose products changed gradually, the intensity of the 2θ = ~17.0, ~22.0 reflections decreased and a broad reflection in the 2θ ranging from 5.0 to 10.0 appeared.

### 2.6. CP/MAS ^13^C-NMR spectra

The acetylation reaction of cellulose was also characterized by CP/MAS ^13^C-NMR spectroscopy. Figure 10 shows the CP/MAS ^13^C-NMR spectra of both native cellulose (spectrum a) and acetylated sample 7 (spectrum b) and sample 22 (spectrum c). In the three spectra, the noticeable signals in the region between 50 to 110 ppm are mostly attributed to the different carbons of cellulose. The resonance line at 106.0 ppm is assigned to the C-1. The peaks at 89.8 and 85.2 ppm are related to the C-4 of crystalline cellulose and disordered cellulose, respectively. A similar trend can be seen in the signal to C-6, namely, the signal at 65.8 ppm is attributed to the crystalline cellulose and the peak at 63.1 ppm is assigned to disordered or crystal surfaces cellulose. In this region there were also two peaks at 73.5 and 75.6 ppm which belong to the C-2−C-5. In addition, the two representative peaks at 171.8 and 21.5 ppm in spectra b and c, which belong to CO and CH_3_ in acetyl group, indicated the occurrence of acetylation reaction. On the other hand, the intensity of the signals at 89.7 ppm for crystalline C-4 and 65.9 ppm for crystalline C-6 decreased sharply in spectra b and c, which reveal that the crystalline structure of the cellulose was disrupted and the acetylation reaction occurs in the system. Finally, the increase of the intensity for peaks at 171.8 ppm (CO) and 21.5 ppm (CH_3_) and the decrease of strength for peaks at 73.5, 75.6 ppm (C-2−C-5) and 65.8 (C-6) in spectra b and c indicated that the extent of acetylation for sample 22 is higher than that of sample 7, which is consistent with the trend of the DS and yield in the Table 1.

In addition, the hydroxyl groups in 6-position should be attacked first by acetyl groups, which is proved by the decreasing intensity of C-6 resonances. The hydroxyl groups in 2-position are closer to the (*O*)-link, and more exposed than the hydroxyl groups in 3-position, thus the hydroxyl groups in 2-position is acetylated more easily than 3-positon. Apparently, in spectra c the peak representing C-1 of CTA has a rupture which may be duo to the oligomers crystalline into the CTA lattice during the reaction. As is known, the higher temperature and the longer time of microwave irradiation result in the hydrolysis of the ester groups and/or the decomposition of cellulose backbones, which produced the oligomers whose number of carbons, are from 2 to 9. The solid-state ^13^C chemical shifts of the oligomers of CTA have been reported in some literatures [30,31].

## 3. Conclusions

The results obtained from our study suggested that microwave-assisted acetylation of cellulose with acetic anhydride in the presence of iodine as a catalyst is a promising method to produce cellulose acetates in a solvent-free system, and that the production of CA in a more economical and environmentally friendly via minimization of energy consumption, solvent, and amount of catalyst quantities, and especially, by minimizing toxicity, may be possible. The microwave assisted time and temperature had an prominent influence on the extent of acetylation, and the DS had an increase trend with the increase amount of iodine, but the effect of the microwave output is not significant, In our work the optimum condition was as following: the cellulose acetate was produced at the reaction time for 30 min at 130 °C with the microwave power 400 W using the 15 mol% iodine.

## 4. Experimental

### 4.1. Materials and reagents

Cellulose samples were obtained from *Caragana korshinskii*, all the water used was distilled. Acetic anhydride, iodine, sodium thiosulfate, ethanol, sodium hydroxide, hydrochloric acid (HCl) were analytical reagents. All chemicals were used without further purification.

### 4.2. The isolation of cellulose

**Figure 11:**
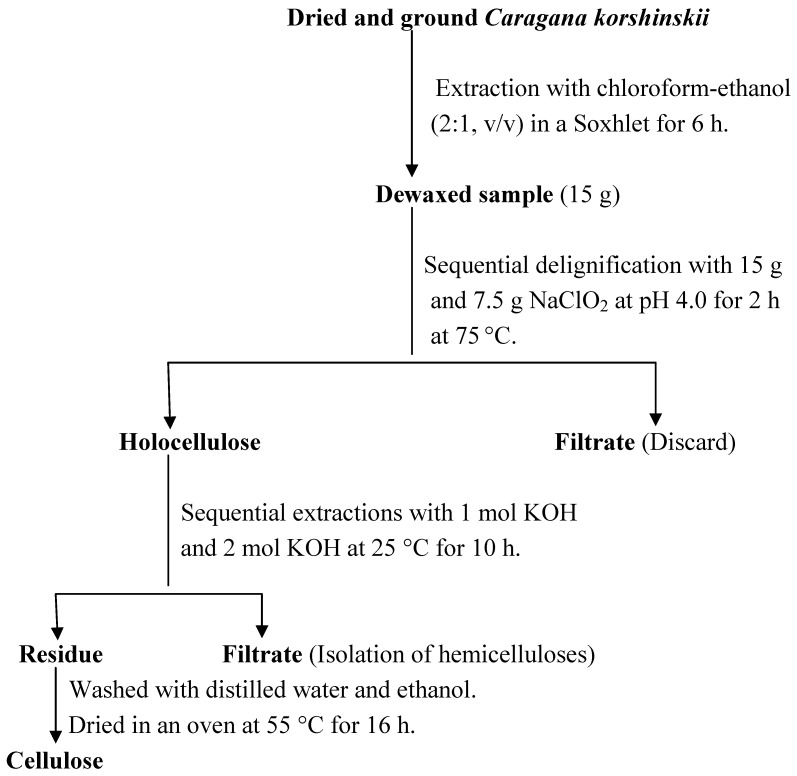
Scheme for isolation of cellulose from *Caragana korshinskii.*

A scheme for isolation of cellulose from *Caragana korshinskii* is shown in Figure 11. The dried and ground *Caragana korshinskii* was first extracted with chloroform-ethanol (2:1, v/v) in a Soxhlet extractor for 6 h so as to remove the extractable materials such as wax. The dewaxed *Caragana korshinskii* was delignified with two sequential processes as the follows: the dewaxed sample (15 g) was soaked in distilled water (300 mL) and sodium chlorite (15 g, solid, 98%) was added into the suspension, the mixture was acidified to pH 4.0 with acetic acid and heated in water bath at 75 °C for 2 h, then sodium chlorite (7.5 g) was added again and the mixture was also acidified to pH 4.0 with acetic acid, heated in water bath at 75 °C for another 1 h. The residue was collected by filtration, subsequently washed with distilled water and 75% (v/v) ethanol, then oven dried at 55 °C for 16 h. The holocellulose obtained was soaked in distilled water with a solid-to-liquor ratio of 1:20 (g·mL^-1^), treated with KOH (1 mol) at 25 °C for 10 h. After filtration, the residue was washed with distilled water and 75% (v/v) ethanol and then oven dried at 55 °C for 16 h. After that, the dried filtration residue was subjected to further extraction with KOH (2 mol) at 25 °C for 10 h. The degree of polymerization (DP) and the crystallinity (I_c_) were 158 and 58.6%, respectively.

### 4.3. Acetylation of cellulose

A mixture of acetic anhydride (10 mL), cellulose (0.4075 g) and different concentrations of iodine (1, 3, 5, 8, 10, 15 mol%; molar ratio of I_2_/AGU) was placed in a 50 mL three-necked flask fitted with a mechanical stirrer. When the mixture was uniformly mixed, the three-necked flask was placed into the microwave oven (XH100B, China) equipped with a reflux condenser and a fiber optic temperature probe. The mixture was heated to 80, 90, 100, 110, 120, and 130 °C and left for 5, 10, 15, 20, 25, 30, and 40 min under an irradiation power of 300, 400, 500, 600, 700, and 800 W. When the reaction was finished, the flask was removed from the microwave oven, and cooled to room temperature. A saturated sodium thiosulfate solution (1–2 mL) was added to the flask and stirred until the mixture color changed from dark brown to colorless, indicating the transformation of iodine to iodide. And then the mixture was transferred to a beaker containing ethanol (30 mL), stirred for several minutes, and filtered. The residue was thoroughly washed with 75% (v/v) alcohol to remove the unreacted acetic acid and byproducts. Samples were dried in a vacuum oven at 60 °C for 12 h. The oven-dried materials were weighed to determine the yield on the basis of initial dry cellulose (Yield (%) = Weight gain/original weight × 100). 

### 4.4. Structural characterization

The determination of DS was conducted by back titration method, which is according to [32,33,34]. Fourier transform infrared (FT-IR) spectra of the native cellulose and acetylated cellulose samples were obtained with dried powdered on the Tensor 27 (Bruker, Germany) in the range of 4,000-400 cm^-1^. Pellets were prepared from mixtures of the samples and KBr (1:100 in weight). 32 scans were accumulated at a resolution of 2 cm^-1^.

The analysis of the native cellulose and acetylated cellulose samples were performed using thermogravimetric analysis (TGA) and differential thermal analysis (DTA) on a simultaneous thermal analyzer (DTG-60, Shimadzu, Japan). The apparatus was continually flushed with nitrogen. The sample were weighed between 8 to 13 mg and heated from the ambient temperature to 600 °C in aluminum pan at a constant heating rate of 10 °C /min.

X-rays diffraction patterns of the native cellulose and acetylated cellulose samples were obtained using a XRD-6000 (Shimadzu, Japan) with a Ni filter and Cu K radiation from 5°-40°. Solid-state cross polarization magic angle spinning carbon-13 nuclear magnetic resonance (CP/MAS ^13^C-NMR) spectra were recorded using a Bruker DRX-400 spectrometer operated at 100 MHz with 5 mm MAS BBO probe at ambient temperature. The delay time 2 s, the acquisition time 0.034 s, the CP contact time 150 μs, the proton 90°, pulse time 4.85 μs and the rotation speed of the sample 6,000 Hz.

## Figures and Tables

**Table 1:** The DS and yield of acetylated cellulose obtained under various microwave irradiation conditions by using 5 mol% iodine as a catalyst.

Acetylation conditions			Acetylated cellulose		
T ( °C )^a^	t (min)^b^	P (w)^c^	Sample No.	DS	Yield (% dry weight)^d^
120	15	300	1	1.4	22
120	15	400	2	1.5	23
120	15	500	3	1.4	22
120	15	600	4	1.4	22
120	15	700	5	1.4	22
120	15	800	6	1.4	7
80	15	400	7	0.78	13
90	15	400	8	0.84	12
100	15	400	9	1.2	16
110	15	400	10	1.1	21
120	15	400	11	1.4	21
130	15	400	12	1.6	25
130	5	400	13	1.5	26
130	10	400	14	1.6	27
130	20	400	15	1.7	22
130	25	400	16	1.8	26
130	30	400	17	1.8	35
130	40	400	18	1.9	46

^a^ Reaction temperature; ^b^ Reaction time; ^c^ Power of microwave oven; ^d^ % increase weight of the sample (g/g).

**Table 2:** The DS and yield of the acetylated cellulose obtained with different concentrations of iodine as a catalyst.

Sample No.	19	20	21	22	23	24
**Iodine(%)^a^**	1	3	5	8	10	15
**DS**	0.48	1.1	1.8	2.0	2.3	2.8
**Yield (% dry weight)^b^**	−	7.2	35.3	37.4	43.6	78

^a^ Molar ratio of I_2_/AGU; ^b^ % increase weight of the sample (g/g)
